# Rab11 Helps Maintain Apical Crumbs and Adherens Junctions in the Drosophila Embryonic Ectoderm

**DOI:** 10.1371/journal.pone.0007634

**Published:** 2009-10-28

**Authors:** Jeremiah F. Roeth, Jessica K. Sawyer, Daniel A. Wilner, Mark Peifer

**Affiliations:** 1 Department of Biology, University of North Carolina at Chapel Hill, Chapel Hill, North Carolina, United States of America; 2 Lineberger Comprehensive Cancer Center, University of North Carolina at Chapel Hill, Chapel Hill, North Carolina, United States of America; University of Texas MD Anderson Cancer Center, United States of America

## Abstract

**Background:**

Tissue morphogenesis and organogenesis require that cells retain stable cell-cell adhesion while changing shape and moving. One mechanism to accommodate this plasticity in cell adhesion involves regulated trafficking of junctional proteins.

**Methodology/Principal Findings:**

Here we explored trafficking of junctional proteins in two well-characterized model epithelia, the *Drosophila* embryonic ectoderm and amnioserosa. We find that DE-cadherin, the transmembrane protein of adherens junctions, is actively trafficked through putative vesicles, and appears to travel through both Rab5-positive and Rab11-positive structures. We manipulated the functions of Rab11 and Rab5 to examine the effects on junctional stability and morphogenesis. Reducing Rab11 function, either using a dominant negative construct or loss of function alleles, disrupts integrity of the ectoderm and leads to loss of adherens junctions. Strikingly, the apical junctional regulator Crumbs is lost before AJs are destabilized, while the basolateral protein Dlg remains cortical. Altering Rab5 function had less dramatic effects, not disrupting adherens junction integrity but affecting dorsal closure.

**Conclusions/Significance:**

We contrast our results with what others saw when disrupting other trafficking regulators, and when disrupting Rab function in other tissues; together these data suggest distinct mechanisms regulate junctional stability and plasticity in different tissues.

## Introduction

Cell-cell adhesion is critical both for development and disease (reviewed in [1]–[4]). A fundamental understanding of cell-cell adhesion will provide insight into the regulation of tissue organization, maintenance, and remodeling during embryonic development and adult homeostasis. The basic knowledge gained from these studies can then be applied to help understand wound healing, tumor metastasis, and organogenesis/tissue engineering.

Adherens junctions (AJs) are critical to cellular adhesion, and are initiated by homophilic interactions between classical cadherins on adjacent cells. Cadherin cytoplasmic domains are organizing centers for connections with cell signaling pathways and the cytoskeletal infrastructure. The core components of AJs are cadherins, β-catenin (βcat-or its fly homolog Armadillo (Arm)), and α-catenin (reviewed in [5]). Arm links the cytoplasmic domain of cadherin to α-catenin, which in turn provides a functional linkage to the actin cytoskeleton. There is currently controversy about whether this linkage is direct, indirect through other actin binding protein partners, or involves cadherin-independent interaction of α-catenin with actin [6], [7]. Clustering of cadherin contacts strengthens adhesion, while cadherin endocytosis is thought to weaken adhesion. In epithelial tissues cadherin-catenin complexes preferentially localize at the apical end of the lateral cell membranes, forming an adhesive belt connecting a cell to its epithelial neighbors. This apical targeting of AJs also helps to establish and maintain epithelial cell polarity. The past 15 years of work yielded a static picture of the protein complexes that participate in these events. One current challenge is to explore the dynamic regulation of AJs as they respond to or initiate changes in the cytoskeleton that mediate cellular morphogenesis.

AJs must maintain a high level of plasticity to allow rapid changes in cellular adhesion to accommodate cell shape changes during morphogenesis. This plasticity likely takes multiple forms, one of which is regulated delivery of cadherin-catenin complexes to remodeling AJs and regulated uptake by endocytosis (reviewed in [8]–[10]). Polarized mammalian cell culture systems (predominantly MDCK cells) have been used to examine regulation of AJ trafficking (reviewed in [9]). In this system, E-Cadherin is specifically targeted to basolateral membranes, where it forms cell-cell adhesive contacts within a monolayer. These studies resulted in a model where intracellular transport of E-Cadherin is an essential determinant of cellular adhesion. Several cellular factors participating in subcellular targeting and subsequent recycling of E-Cadherin were identified, and include signaling molecules, small GTPases, cytoskeletal components, and proteins involved in endocytosis (reviewed in [8]–[10]). However, the exact regulatory mechanisms controlling these processes are not yet clear, and how regulation of E-Cadherin trafficking contributes to tissue organization and homeostasis in an intact animal is poorly understood, though recent advances have been dramatic.

Endocytosis of classic cadherins like E-cadherin is likely to largely occur through the clathrin-mediated pathway (reviewed in [8]–[10]). Once internalized, cadherins accumulate in Rab5-positive early endosomes. From there they can be directed via a Rab7-mediated pathway to late endosomes and ultimately lysosomes for destruction. Alternately, they can be recycled to the cell surface via the Rab11-positive recycling endosome. The Rab11-recycling endosome has also been implicated in the initial targeting of newly synthesized E-cadherin to the basolateral plasma membrane, both in cultured mammalian cells [11], [12] and in the *Drosophila* pupal epidermis [13]. In the latter case, at least, it acts in concert with the exocyst complex.

One mechanism of regulating cadherin trafficking involves interactions with catenins. βcat is required for cadherin delivery to the cell surface in cultured MDCK cells [14] and *Drosophila* embryos [15]. Its distant relative p120 is also a key regulator of cell adhesion. Vertebrate *p120* is an essential gene with roles in many tissues, and regulates stability of cadherins at the cell surface [16]–[18], by regulating cadherin endocytosis and degradation [16], [18]–[20], and also escorting E-cadherin during kinesin mediated movement to the cell surface [21]. *Drosophila* and *C. elegans* p120 positively regulate cell adhesion when cadherin levels are reduced [22]–[24]. Rho family GTPases also regulate AJ stability, perhaps via trafficking (reviewed in [25]). During *Drosophila* embryogenesis, Rho mutants or dominant negative Rho disrupt cadherin localization [26], [27]. Another small GTPase, Rap1, may also regulate cadherin trafficking [28]–[30].

A number of studies have begun to explore how AJ and other junctional proteins are trafficked during morphognesis, implicating Rab5, Rab11, and some of their partners, with different players targeting distinct junctional proteins at different times and places. For example, in the *Drosophila* imaginal disc epithelium, inactivation of either Rab5 or the syntaxin Avalanche lead to failure to endocytose the apical junctional protein Crumbs (Crb), disrupting epithelial architecture [31]. Surprisingly, in this tissue DE-cadherin (DEcad) accumulation at the membrane is unaltered [31], although in the embryo Crb is essential for AJ maintenance [32]. In the developing wing imaginal disc epithelium, DEcad is trafficked through both Rab5-positive early endosomes and Rab11-positive recycling endosomes, in a process that is dynamin, Rab11, and exocyst dependent [33]. This regulated cadherin recycling appears to be required for junctional remodeling during planar polarization in this tissue. Finally, in the developing embryonic ectoderm, removing function of the exocyst protein Exo84 leads to loss of Crb localization, which is followed later by loss of AJ proteins [34].

Through analyses like these, it has become clear Rab11 plays a wide variety of roles in *Drosophila* embryonic and postembryonic development, not all of which are confined to polarized epithelial cells. For example, Rab11 was first found to be critical for polarization of *Drosophila* oocytes [35], [36], and in the early embryo, it is critical for membrane trafficking during syncytial nuclear divisions [37] and cellularization [38]. In highly polarized photoreceptor cells, Rab11 regulates polarized traffic of membrane proteins. Rab11, together with Myosin V, Rip11, and the exocyst direct polarized apical secretion of rhodopsin and other apical proteins [39]–[41]. In at least two cell types, the female germline stem cells [42] and the pupal eye imaginal disc [43], loss of Rab11 disrupts the surface accumulation of AJ proteins.

This raises the possibility that regulated endocytosis of junctional proteins might influence morphogenesis. Consistent with this, the transcription factor Ribbon upregulates apical Rab11 and maintains apical accumulation of the polarity protein Crumbs (Crb) during elongation of *Drosophila* salivary glands [44]. An even more striking example comes from morphogenesis of *Drosophila* tracheal tubes, where scientists traced the pathway from secreted cell signal to morphological outcome [45]. Tracheal cells form tubes, in part, by cell intercalation, but this needs to be restricted to the right time and place. The signaling molecule Wingless upregulates the transcription factor Spalt and inhibits cell intercalation in the dorsal trunk of the trachea. Spalt upregulates Rab11-mediated recycling of DEcad, by regulating levels of Rab5′s partner Rip11 [45] This elevates surface DEcad, presumably increasing cell adhesion and blocking intercalation specifically in these cells. Regulation of morphogenesis by regulating cadherin trafficking is also important during vertebrate development. For example, Wnt11 is a key regulator of zebrafish gastrulation. It acts in part by promoting the activity of Rab5, and thus upregulating the endocytosis of E-cadherin in mesendoderm cells [46].

We explored DEcad trafficking in the ectoderm of *Drosophila*, a powerful model to study regulation of cell adhesion and cell shape changes in an intact animal. During embryogenesis, specific ectodermal cells undergo coordinated shape changes to accommodate complex morphogenetic events such as invagination of neural precursors, extension and retraction of the germband, dorsal closure and head involution. This same field of cells must also rearrange cell contacts in response to cell division, a process that also requires AJ remodeling. Both morphogenetic movements and cell division patterns are well characterized and are tightly regulated during normal development. Thus, this epithelial tissue provides an excellent model to identify and examine the specific molecular interactions between AJ components and their regulatory factors. Further, previous work makes it clear different subpopulations of cells within the ectoderm are differentially sensitive to reduction in DEcad. The ventral neuroectoderm, from which neural precursors delaminate, is much more sensitive to reduced levels of DEcad than is the dorsal ectoderm, and eliminating neuroblast invagination eliminates this difference [47]. Further the ventral ectoderm is also more sensitive to loss of function of cdc42, which normally restrains endocytosis of the apical junctional protein Crb, and thus stabilizes AJs [48].

To characterize the role protein trafficking plays in AJ remodeling during embryogenesis, we focused on Rab family GTPases. To test if Rab11 regulates trafficking of junctional proteins, we used two methods to disrupt Rab11 function. First, we used the GAL4-UAS system to express dominant-negative Rab11 (Rab11DN) in specific places and times. This had dramatic effects, triggering fragmentation and ultimate loss of AJs. This was preceded by the loss of Crb from the apical domain. In contrast, the basolateral protein Dlg was unaffected. To control for off-target effects of Rab11DN, we used an embryonic lethal *rab11* allele (*rab11^j2D1^*). This also led to fragmented AJs, leading to defects in epithelial integrity. Phenotypes of embryos with reduced Rab11 function were similar to those of *crb* mutants [32], supporting the idea that Rab11 regulates Crb trafficking in this tissue. We also explore Rab5 in the ectoderm and amnioserosa.

## Results

### A subset of ectodermal DEcad localizes to vesicles that may be trafficking intermediates

**Figure 1:**
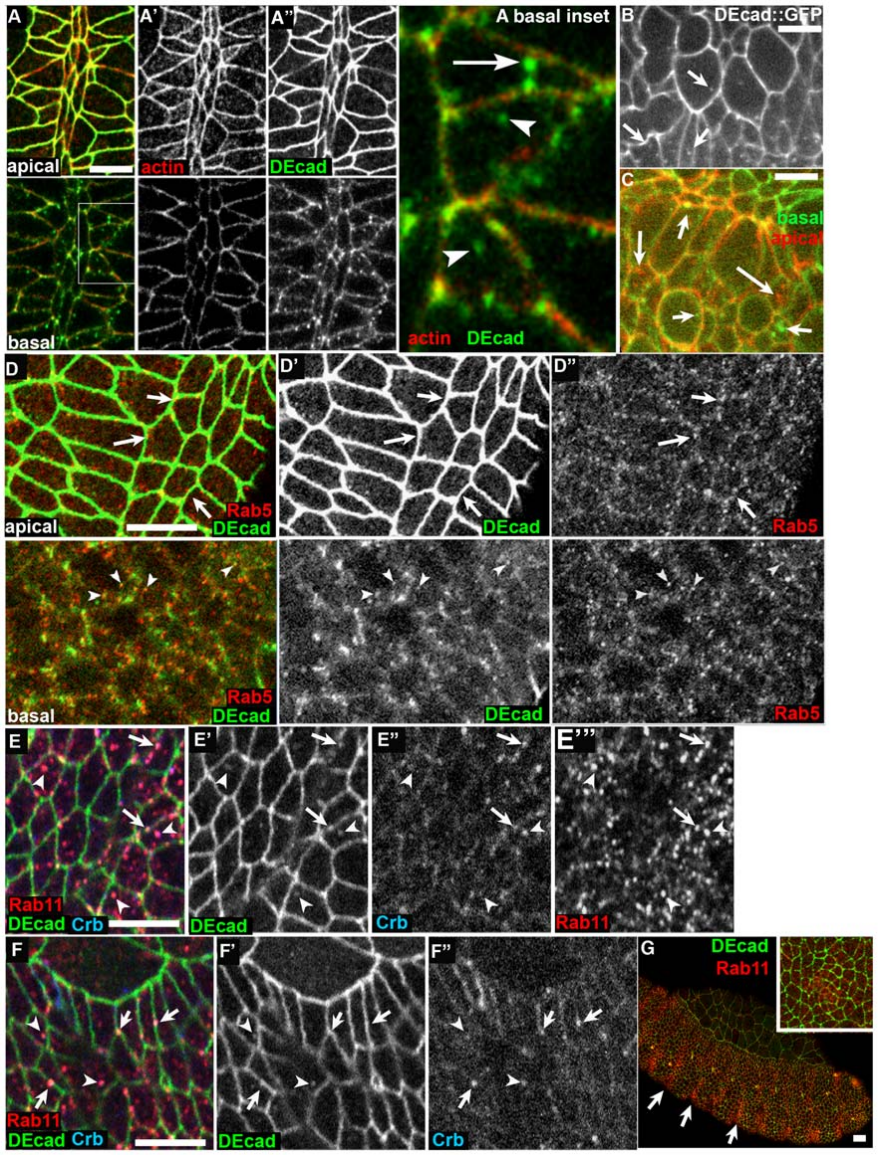
DEcad trafficking in the ectoderm. Wild type embryos, antigens indicated. A–D. ventral ectoderm, Stage 9. E–G. Lateral and dorsal ectoderm, Stage 13. A–C. DEcad is in putative vesicles enriched in the basal part of the cell. A. Ventral midline, anterior up. Apical and basal planes. Box in basal plane is shown enlarged in image at right. Arrow, DEcad punctum at cortex. Arrowheads, putative cytoplasmic vesicles. B,C. Stills from a video of DEcad-GFP in ventral ectoderm. C shows two planes of the same image, with the apical plane red and the basal plane green. Note both cortical and cytoplasmic putative vesicles (arrows). D. DEcad can localize to Rab5-positive putative vesicles. Apical and basal planes. In the apical plane Rab5 puncta are enriched near the AJ (arrows). In the basal plane, Rab5 is in cytoplasmic puncta, a subset of which contain DEcad (arrowhead). E,F. Rab11 localizes to both cortical (arrows) and cytoplasmic (arrowheads) puncta, some of which colocalize with DEcad and Crb. G. Rab11 puncta are enriched in segmental groove cells (arrows) and in cells invaginating to form trachea (inset). Scale bars = 15 µm.

To begin exploring the role of DEcad trafficking in the epidermis, we carried out preliminary studies of DEcad localization in the ectoderm of wild-type embryos. While the majority of DEcad localizes to apical AJs (Fig. 1A–A″, apical), in the basal part of the cells we detected a subpopulation of DEcad that localizes to punctate structures (Fig. 1A–A″, basal). Some of these are cortical (e.g., Fig. 1A, basal inset, arrow), while others are intracellular (Fig. 1A, inset, arrowheads). We could also observe this vesicular population of DEcad in living embryos expressing DEcad-GFP (Fig. 1B,C, Video S1). Interestingly, these puncta appear to be particularly apparent in cells undergoing cell shape changes, such as during the formation of the ventral midline or in cells whose neighbors are undergoing cell division, when we hypothesize AJs would be actively remodeled; quantitation of vesicle number would be useful to test this hypothesis. We hypothesize these structures are vesicular trafficking intermediates resulting from the active remodeling of AJs.

To test the hypothesis that these are vesicular trafficking intermediates, we explored whether they co-localize with markers of early (Rab5) or late (Rab11) endosomes. In the embryonic ectoderm, Rab5 localizes to punctate putative vesicles. A subpopulation is localized along the cell cortex at the level of AJs (Fig. 1D–D″ apical, arrows), while others are found in the basal part of these cells (Fig. 1D–D″, basal). A small subset of DEcad puncta colocalize with Rab5-positive vesicles (Fig. 1D–D″, basal, arrowheads), consistent with the idea that DEcad trafficks through the early endosome in the ectoderm.

We also examined localization of Rab11, which marks the recycling endosome [49]. Wild-type Rab11 also localizes to punctate putative vesicles in the ectoderm (Fig. 1E–F″). A subset of these overlap AJs (Fig. 1E–F″, arrows). Another subset of the Rab11-positive non-cortical vesicles co-localize with DEcad (Fig. 1E–F″, arrowheads). Intriguingly, some of these vesicles are also positive for a different apical membrane protein, Crumbs (Crb; Fig. 1E″,F″). In analyzing these images, we noted Rab11-positive putative vesicles appear to be enriched in certain ectodermal cells undergoing shape change, including segmental groove cells (Fig. 1G, arrows), and cells invaginating to form tracheal pits (Fig. 1G, inset). Others found subcortical Rab11 enrichment in epidermal and amnioserosal cells during dorsal closure [50]. Of course, apical constriction might result in the same number of vesicles being present in a reduced apical area, leading to an appearance of Rab11 enrichment.

**Figure 2:**
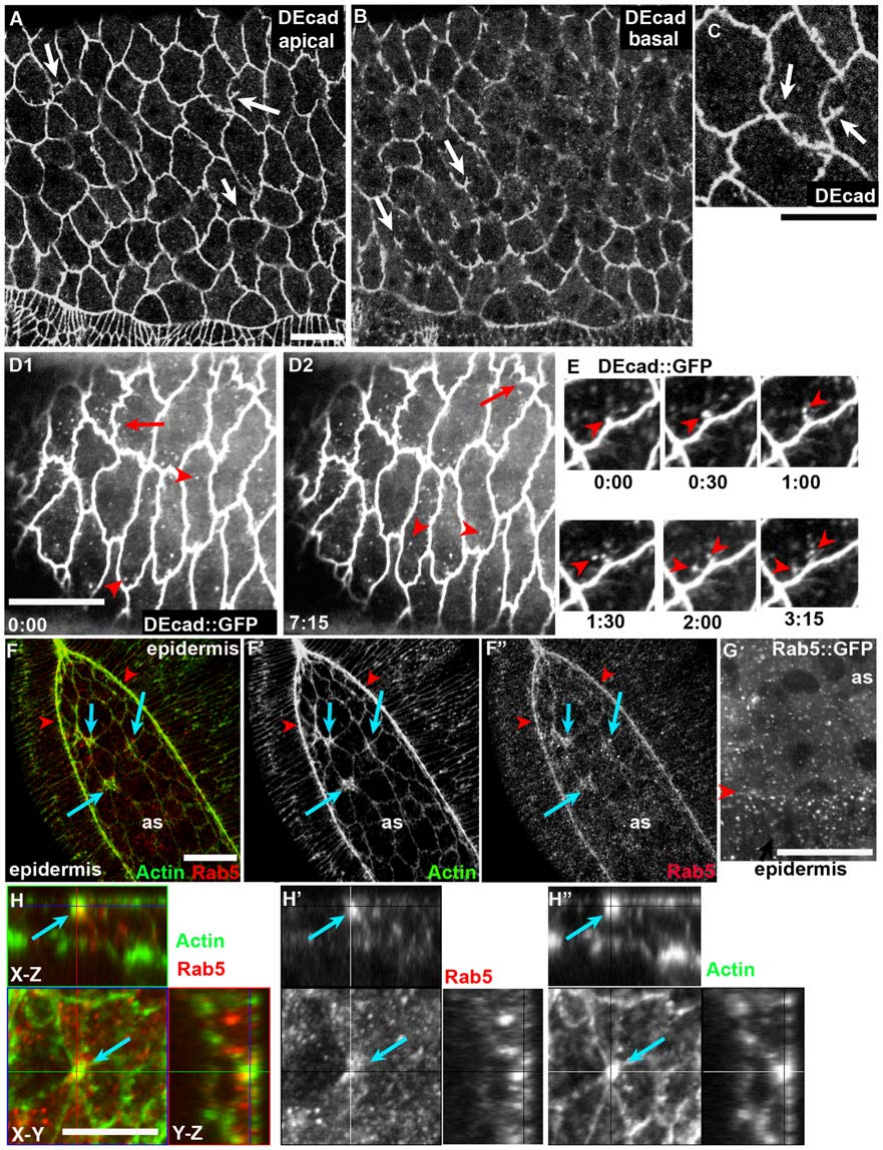
DEcad trafficking in the amnioserosa. Wild type embryos, stage 13–14, dorsal views, antigens indicated. A–C. DEcad in the amnioserosa. C is a close-up of A. In addition to AJ localization, DEcad is also localized to tubular invaginations (arrows), which are present apically and also to a lesser extent on the basolateral membrane D,E. Video stills of embryos expressing DEcad-GFP. D. Tubular invaginations (arrows) and putative vesicles (arrowheads). E. Putative vesicles emerging from a tubular invagination. F. Rab5 appears enriched at the apical ends of rapidly constricting amnioserosal cells (arrows) and at the leading edge (arrowheads) G. Rab5-GFP localizes to putative vesicles that are enriched at the leading edge (arrowhead). H. Actin and Rab5 appear enriched at the apical ends of amnioserosal cells that have completed invagination ahead of their neighbors (arrows). X−Y = dorsal view of apical surface, XZ and YZ, cross-sections. Scale bars = 30 µm.

Another morphogenetically active tissue in the embryo is the amnioserosa (AS). AS cells undergo dramatic cell shape changes during germband extension and during germband retraction. They then undergo apical constriction, helping drive dorsal closure. During this event, these squamous epithelial cells must eliminate large amounts of apical membrane rapidly. In AS cells, DEcad localizes to both AJs (Fig. 2A–D) and to punctate putative vesicles (Fig. 2D, arrowheads, Video S2), as in the ectoderm. DEcad and other junctional proteins also localize to intriguing tubular membrane invaginations (Fig. 2A–D, arrows; data not shown); most of these invaginations are in the apical junctional plane, but some are found more basally (Fig. 2B, arrows). Live imaging of DEcad::GFP suggests that some of these invaginations appear to be the source of internalized vesicles (Fig. 2E). This process of tubular invaginations of DEcad containing membrane as a source of vesicles is intriguingly similar to a process observed in *Cdc42* mutant wing imaginal discs [51].

Amnioserosal cells all apically constrict, but a subset constrict earlier than their neighbors. While Rab5-positive putative vesicles are found in all amnioserosal cells (Fig. 2G), as they are in epidermal cells, Rab5-vesicles appear enriched at the apical ends of cells that have completed apical constriction—this is seen both with endogenous Rab5 (Fig. 2F–F″, arrows) and when wild-type Rab5 is overexpressed in the AS (Fig. 2H–H″). However, once again this may simply reflect the same number of vesicles present in a reduced apical area. There is also apparent enrichment of Rab5 along the leading edge of the epidermis (Fig. 2F,G arrowheads).

### Rab11 inactivation leads to disruption of epithelial integrity

**Figure 3:**
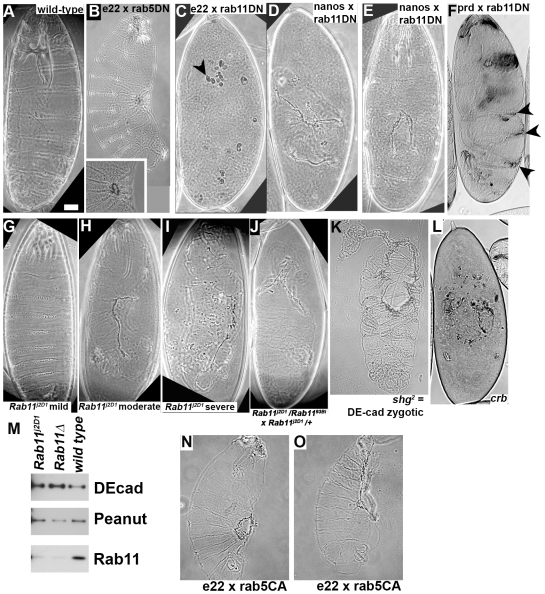
Disrupting Rab11 function disrupts epidermal integrity. A–L, N,O. Cuticles, anterior up, genotypes indicated. B, inset. Close-up of dorsal hole. C. Arrowhead, cuticle fragment. F. Arrowheads, segmentally repeated disruption of the cuticle. Scale bar = 30 µm. M. Immunoblot of embryos of indicated genotypes with Rab11, DEcad and Peanut (Pnt) antibodies—the latter two are loading controls. Homozygous mutant embryos were hand selected using a GFP-marked Balancer chromosome.

These data were consistent with the idea that trafficking of junctional proteins by Rab5 and Rab11 might be important during normal embryonic morphogenesis. To test this hypothesis, we first explored the effects of expressing dominant negative forms of these two Rab proteins on morphogenesis. As an initial screen, we used the embryonic cuticle as an assay. This is secreted by epidermal cells at the end of embryogenesis and provides an excellent read-out of both cell fate choices and the successful completion of morphogenetic movements (Fig. 3A). Ubiquitous expression of a dominant negative Rab5 protein (Rab5 S43N;[52], [53]) using the e22-GAL4 driver did not cause substantial epithelial integrity defects, but did lead to defects in dorsal closure and head involution; cuticles had small dorsal holes and defective head skeletons (Fig. 3B). In contrast, expression of a dominant negative form of Rab11 (Rab11 N124I, a GTP binding mutant; [41], [54]—referred to below as Rab11DN) using a variety of GAL4 drivers led to severe disruption of the cuticle. Ubiquitous zygotic expression using the e22-GAL4 driver led to fragmentation of the cuticle into small pieces (Fig. 3C, arrowhead). This resembles the cuticle phenotype of loss of maternal and zygotic DEcad or Arm [15], [47] or zygotic loss of the apical regulator Crb (Fig. 3L;[55]). Maternal expression of Rab11DN using nanos-GAL4 also resulted in disruption of the epidermis, leading to holes in the cuticle (Fig. 3D,E). Expression of Rab11DN using prd-GAL4 , which expresses in every other body segment, resulted in segmentally periodic disruption of cuticle integrity (Fig. 3F, arrowheads).

This led us to explore the effects of Rab11 further. To assess whether the disruption of the cuticle we observed was due to Rab11 loss-of-function and not due to off-target effects of the dominant negative construct, we characterized the phenotypes of two *Rab11* mutants. Removal or strong reduction of maternal Rab11 leads to defects in oogenesis or syncytial divisions [35], [37], precluding analysis of morphogenesis. We characterized *Rab11^j2D1^*, a P-element insertion into the gene. This mutation is a strong hypomorph, as it reduced the levels of Rab11 protein as assessed by immunoblotting of embryonic extracts from homozygous mutant embryos (Fig. 3M; homozygous mutants were selected using a GFP marked Balancer chromosome—the control is a *Rab11* deletion allele *Rab11^ΔFRT^*; [42]). *Rab11^j2D1^* is embryonic lethal with high but not complete penetrance (15.9% of the progeny of heterozygous parents vs. the 25% expected for complete lethality). Most dead embryos secrete cuticles with defects in head involution and holes in the ventral cuticle, consistent with the idea that reduction in Rab11 function impairs cuticle integrity (Fig. 3G–I). These resembled in severity both Rab11DN driven by nanos-GAL4 (Fig. 3D,E), and embryos zygotically mutant for *shotgun (shg)*, the gene encoding DEcad (Fig. 3K). In the most severe cases, portions of the cuticle of *Rab11^j2D1^* mutants were reduced to fragments (Fig. 3I), similar to, though less severe than, those in embryos lacking zygotic Crb (Fig. 3L). We also generated embryos whose mothers were transheterozygous for two different Rab 11 alleles (*Rab11^j2D1^*/*Rab11^93Bi^*) and crossed them to males heterozygous for *Rab11^j2D1^*. Consistent with previous work examining cellularization [56], many embryos showed complete failure early in embryogenesis and failed to produce cuticle (data not shown). A subset of progeny died with large holes in their cuticles (Fig. 3J); the fraction that did so was consistent with these being those not paternally rescued by wild-type *Rab11*. Others, using different alleles of *Rab11*, observed defects in cell shape and amnioserosal cell integrity during dorsal closure [50]. Together, these data suggest reducing Rab11 function disrupts the integrity of the epidermis.

### Rab11 inactivation in the ectoderm leads to AJ fragmentation

**Figure 4:**
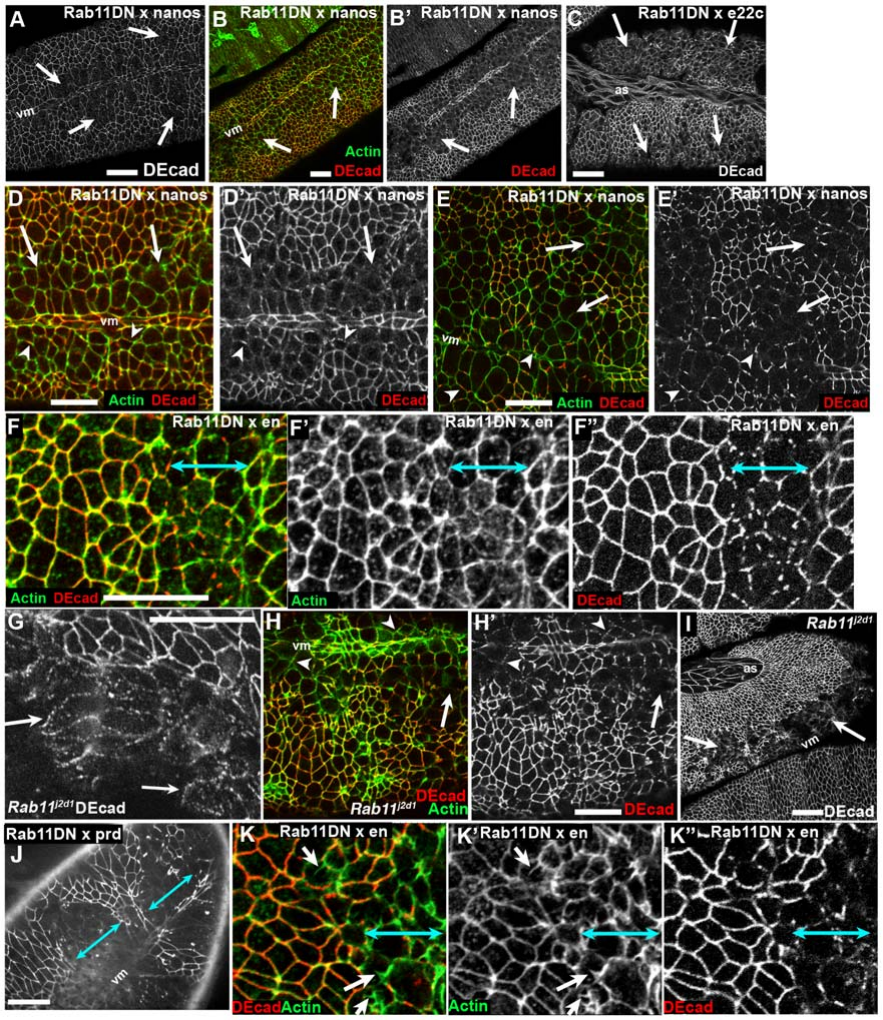
Disrupting Rab11 destabilizes adherens junctions. Embryos, genotypes and antigens indicated. A,B,D,E Ventral views, Stage 9–10. Maternal expression of Rab11DN using nanos-GAL4 destabilizes AJs in the ventralmost ectoderm (arrows), while cortical actin remains intact. C. Stage 10–11. Ubiquitous zygotic expression of Rab11DN beginning at stage 10 using e22-GAL4 has a similar effect. F. Expression of Rab11DN in the posterior compartment using en-GAL4 (blue arrows) destabilizes AJs. G–I. Loss of function mutations in *Rab11* also cause disruption of epidermal integrity. G. *Rab11^j2d1^*. Arrows, fragmented AJs. H. *Rab11^j2d1^*. Arrowheads, fragmented AJs. Arrows, loss of AJs. I. *Rab11^j2d1^*. Arrow, loss of ventral epidermis. J. Expression of Rab11DN in every other segment using prdGAL4 (blue arrows) disrupts AJ localization of Arm-GFP. K. Expression of Rab11DN in the posterior compartment using en-GAL4 (blue arrows) increases apical actin protrusiveness (arrows). Scale bars = 30 µm.

To examine the cell biological effects of reducing Rab11 function, we examined the localization of DEcad. We first expressed Rab11DN ubiquitously using the maternal GAL4 driver nanos-GAL4. The initial stages of gastrulation appeared normal. However, during the extended germband stage, DEcad localization, which should form belt-like AJs around the apical end of each ectodermal cell, began to look abnormal. Junctional DEcad became less continuous, and then was lost. This effect was most striking in the ventral neurectoderm, while the dorsal epidermis was relatively unaffected. Cortical actin remained in affected cells, although they expanded apically, as if they were not able to maintain a columnar shape (Fig. 4A–B′,D–E′). We saw similar effects when we expressed Rab11DN using the ubiquitous zygotic GAL4 driver e22-GAL4 (Fig. 4C; the effects were more pronounced in the posterior germband, which we have seen with other constructs driven with this GAL4 driver). To explore whether this effect was cell autonomous, we used the segmentally restricted driver engrailed (en)-GAL4, expressed in the posterior cells of every segment. Junctional fragmentation was restricted to the expression domain of the driver (Fig. 4F–F″, blue arrows). Expression of Rab11DN in every other segment using prd-GAL4 had a similar effect on DEcad's junctional partner Arm (Fig. 4J; visualized using a functional Arm-GFP fusion).

To examine whether the effects on AJ maintenance were due to off-target effects of the dominant-negative construct, we examined embryos homozygous for a strong loss-of-function allele of *Rab11*, *Rab11^j2D1^*. They also had loss of DEcad, with the ventral ectoderm most sensitive (Fig. 4H), and we observed fragmentation of AJs (Fig. 4G, arrows). After germband retraction (which was sometimes incomplete), we could observe holes in the ventral epidermis (Fig. 4I), consistent with the cuticle phenotype (Fig. 3H,I). Consistent with the idea that this loss of cortical DE-cad was biologically relevant, we found that maternal heterozygosity for a null allele of DE-cadherin (*shg^R69^*) enhanced the embryonic lethality of *Rab11^j2D1^*, increasing lethality from 15.5% (n = 437) to a level near that for complete embryonic lethality (23.5%; n = 391; half of these embryos would also be zygotically heterozygous for *shg^R69^*).

As AJs fragmented, we also saw an additional striking cell biological change. Wild-type cells have elevated cortical actin at the level of AJs and have apical surfaces that are not very protrusive (Fig. 4K–K″, non-expressing cells at left). In contrast, as AJs fragmented, the amount of apical actin was elevated, and the cells accumulated actin in apparent apical protrusions (Fig. 4K–K″, arrows; Rab11-DN expressing cells at right indicated by double-headed arrow). This is consistent with AJs restraining protrusive behavior, something we previously observed when AJs were destabilized by reduction in activity of the formin Diaphanous [57].

### Rab11DN does not affect cortical Dlg but causes Crb loss that precedes loss of DEcad

**Figure 5:**
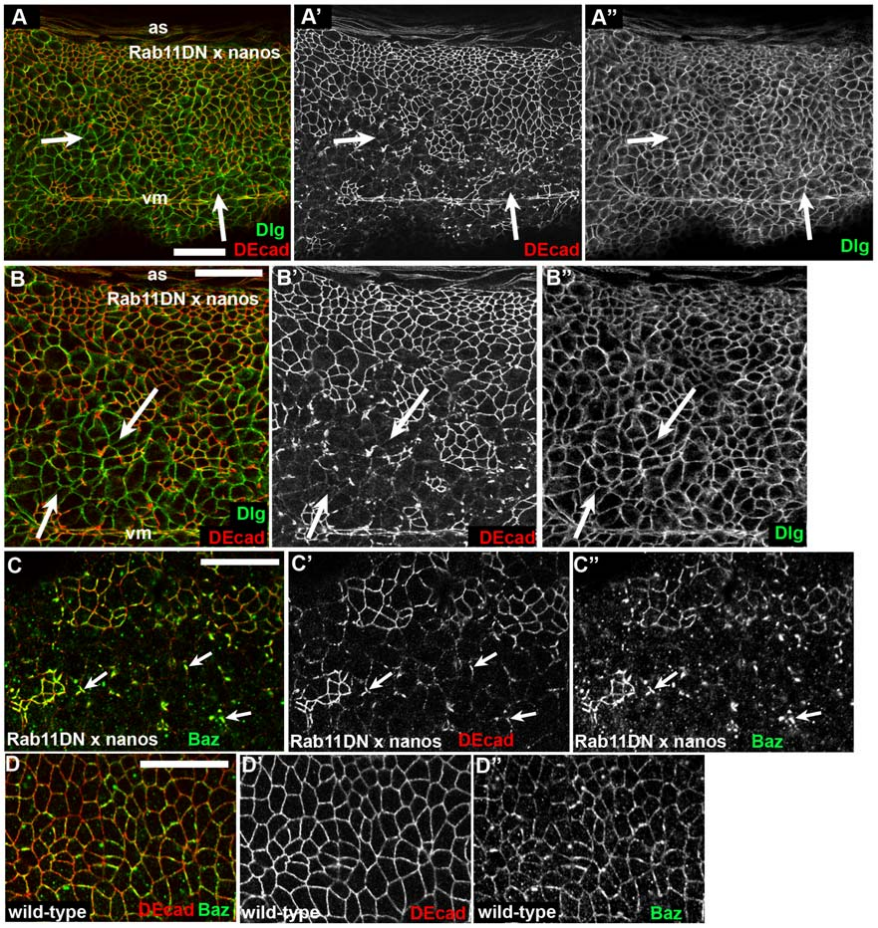
Disrupting Rab11 function does not disrupt basolateral junctions. Stage 9 embryos, ventral views, anterior left, expressing Rab11 maternally using nanos-GAL4. Antigens indicated. B is a close-up of A. Arrows, locations where DEcad localization is disrupted and Dlg localization remains unperturbed. C. Baz loss appears to largely parallel that of DEcad. Arrows, fragments of AJs where residual DEcad and Baz colocalize. D. Wild type control, showing cortical localization and punctate background with Baz antibody. Scale bars = 30 µm.

We next explored whether this effect was specific for AJs, or affected other junctional proteins or polarity regulators. Dlg is a marker of basolateral junctions [58], which localize below AJs. Basolateral junctions appeared to remain intact. Cells that had lost DEcad or in which AJs were fragmented maintained continuous Dlg staining around the cortex, though the apical area increased (Fig. 5A–B″, arrows).

**Figure 6:**
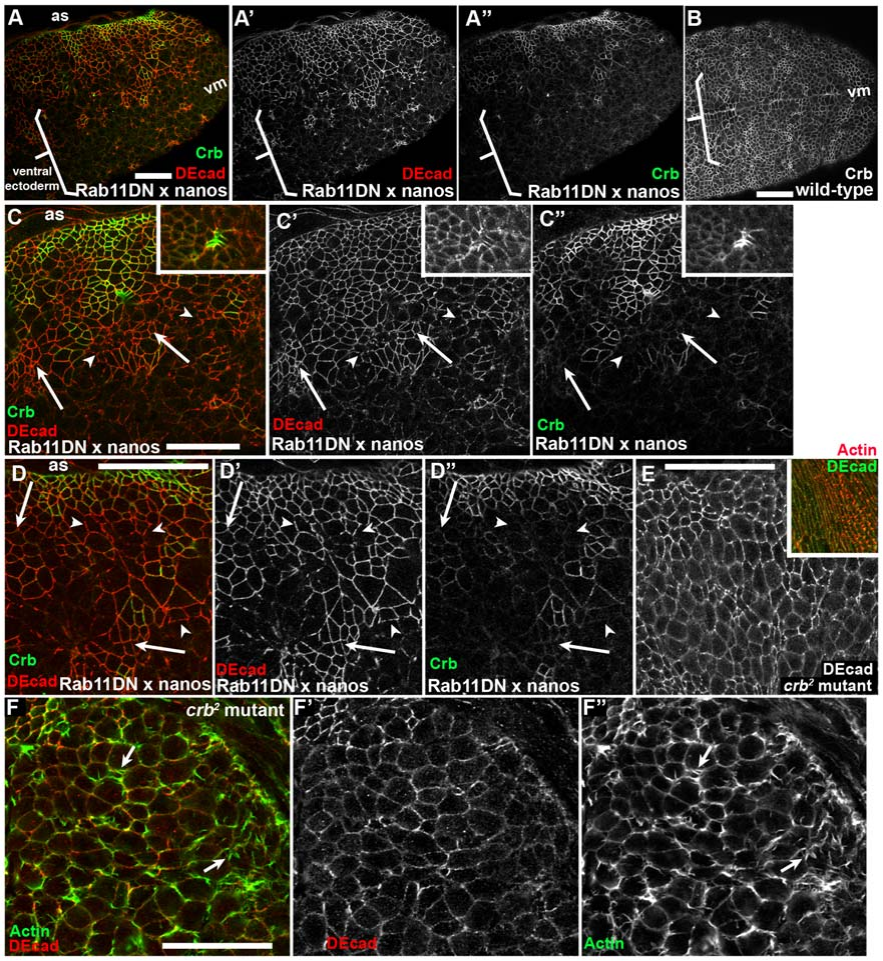
After disrupting Rab11 function, loss of Crb precedes loss of DEcad. Embryos, ventral views, anterior left. Antigens indicated. A,C,D. Stage 9–10 embryos expressing Rab11DN maternally using nanos-GAL4. B. Wild type control. A. Crb is completely lost from the ventral ectoderm (bracket) while some cells still retain cortical DEcad. B. Wild type embryo at the same stage. C,D. Higher magnification views of ventral ectoderm. Arrows, cells with reduced Crb and intact AJs. Arrowheads, cells with greatly reduced Crb and fragmenting AJs. E. Stage 10 *crb^2^* zygotic mutant also showing fragmented AJs. Inset shows stage 14 *crb^2^* mutant, with a patch of intact dorsal epidermis secreting dorsal hairs. F. Stage 11–12 *crb^2^* mutant. Cells with fragmented AJs have elevated apical actin and increased protrusiveness (arrows). Scale bars = 30 µm.

In contrast, there was a striking effect on the junctional localization of the apical membrane regulator Crb. Crb localizes apically to AJs and is required for AJ maintenance after gastrulation [32]. In embryos expressing Rab11DN, Crb was lost from the cortex, with the effect strongest in the ventral ectoderm (Fig. 6A–A″, brackets vs. Fig. 6B). Double labeling of embryos with antibodies to DEcad and Crb revealed that Crb reduction appears to precede reduction of DEcad—many cells with strongly reduced Crb staining retained significant junctional DEcad (Fig. 6C–D″, arrows), and cells with fragmented AJs often had nearly lost Crb (Fig. 6C–D″, arrowheads). At times several cells with small apical ends marked by Crb appeared to be invaginating (Fig. 6C–C″, inset). The fact that loss of Crb after expression of Rab11DN precedes loss of DEcad is consistent with the documented failure to maintain AJs in *crb* mutant embryos [32]—AJ fragmentation in these embryos resembles that in cells expressing Rab11DN (Fig. 6E), including the relative resistance of the dorsal epidermis to loss of Crb (Fig. 6E, inset; actin structures are dorsal hairs). Strikingly, loss of intact AJs in *crb* mutants also correlated with elevated apical actin and increased cell protrusiveness (Fig. 6F–F″, arrows), as we observed in embryos expressing Rab11DN (Fig. 4K). In the future, it would be interesting to further test the hypothesis that Crb is the primary target by exploring genetic interactions between *Rab11* and *crb*.

We also examined another component of apical junctions, Bazooka (Baz = fly Par3). Baz initially localizes with AJs and during mid-embryogenesis takes up a slightly more apical position [59], [60]. Baz is normally cortical (Fig. 5D–D″; the antibody had a punctate background even in wild-type). In embryos expressing Rab11DN, loss of Baz largely paralleled that of DEcad, and Baz and DEcad co-localized in fragmenting AJs (Fig. 5C–C″, arrows).

### Altering Rab5 function has more subtle effects on morphogenesis

**Figure 7:**
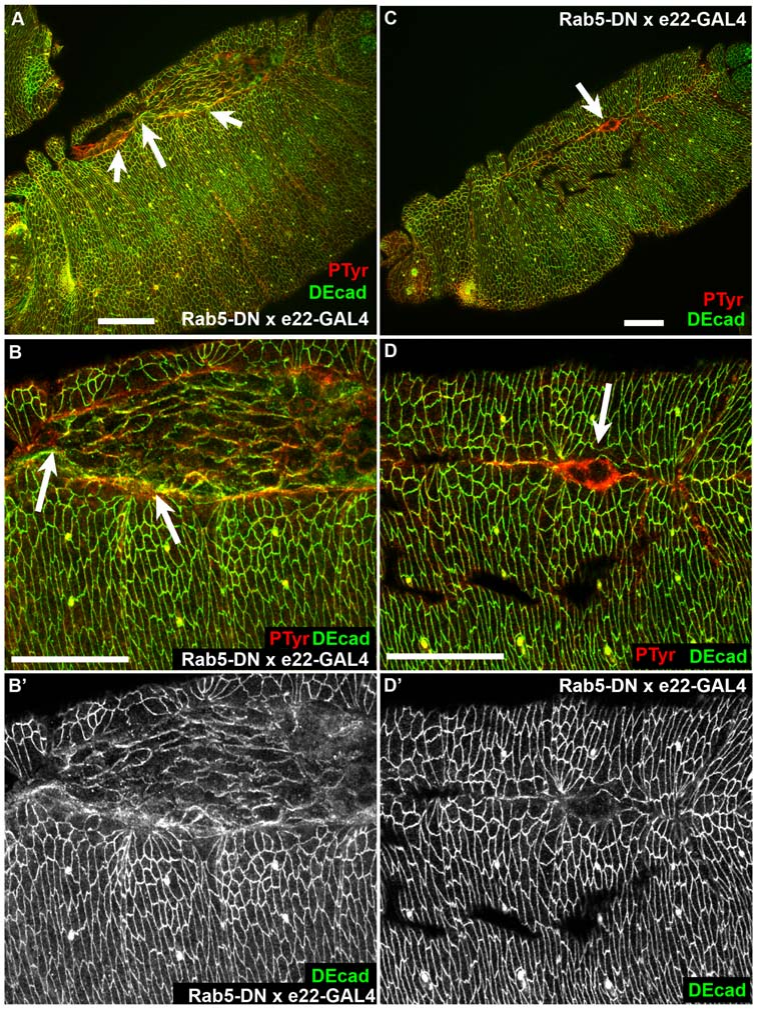
Expressing dominant negative Rab5 disrupts dorsal closure but does not dramatically alter cell surface levels of DEcad. Stage 14–15 embryos expressing Rab5DN under the control of e22-GAL4, antigens indicated. A, B. Low and higher magnification views of same embryo. Arrows, uneven leading edge. C,D. Low and higher magnification views of same embryo. Arrow, dorsal pucker where epidermal sheets did not meet correctly. Scale bars = 30 µm.

Neither Rab5DN nor Rab5CA led to disruption of ectodermal epithelial integrity, as assessed by cuticle preparations (Fig. 3B,N,O). To explore whether they affected DEcad trafficking or had effects on morphogenesis, we examined localization of AJ proteins and cell shape changes in embryos mis-expressing these mutant forms of Rab5. Ubiquitous expression of dominant negative form Rab5 (Rab5 S43N; [52], [53]) led to mild disruption in the completion of dorsal closure (Fig. 3B). This was also seen in fixed embryos stained to reveal DEcad and phosphotyrosine (PTyr), a cortical marker. The normally even leading edge sometimes became uneven (Fig. 7A–B′), and closure often left a dorsal pucker rather than going to completion (Fig. 7C–D′). Dorsal epidermal cells did not appear as elongated as normal, even at the end of dorsal closure. However, despite these defects in morphogenesis, cortical levels of DEcad appeared unchanged (Fig. 7B′,Fig. 7D′′) from wild type or sibling non-expressing controls.

**Figure 8:**
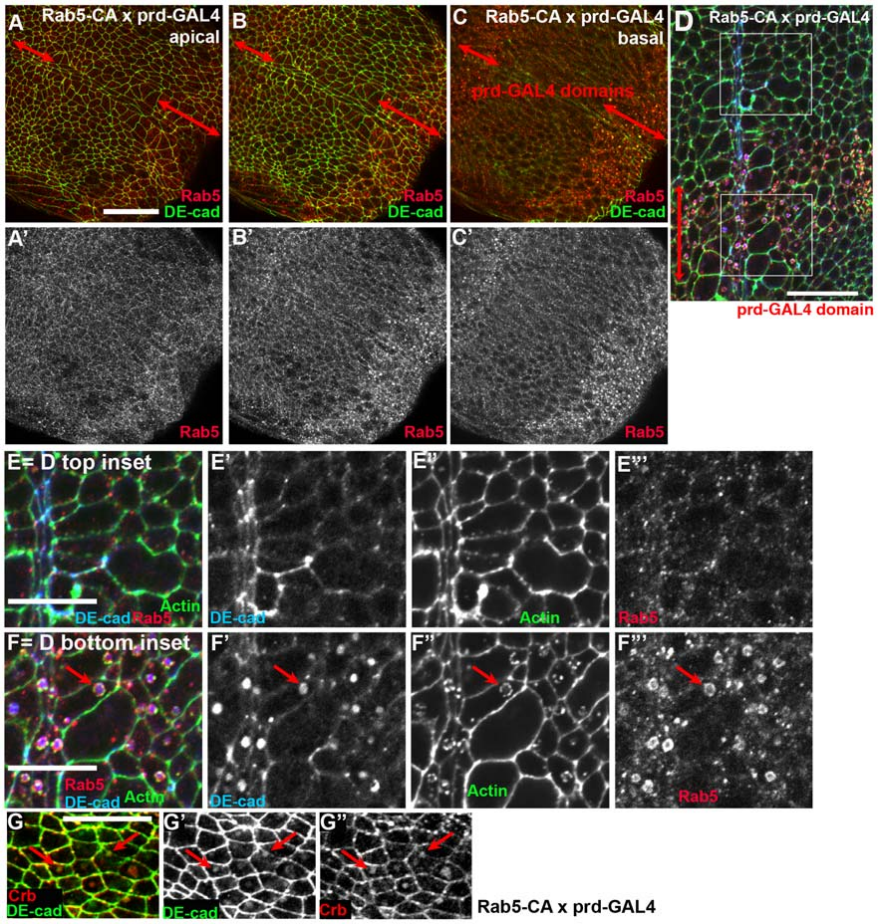
Activating Rab5 leads to DEcad accumulation in enlarged early endosomes. Embryos expressing Rab5CA under the control of prd-GAL4 or e22-GAL4, antigens indicated. A–G. Ectoderm, stage 9–10 embryos. Double-headed arrows, prd-GAL4 expression domain. A–C represent successively more basal sections. E and F are close-ups of boxes in D, representing cells not expressing (E) or expressing (F) Rab5CA. Arrows in F indicate an example of an enlarged endosome. Arrows in G show DEcad and Crb co-localizing in putative vesicles. Scale bars A−D = 30 µm. Scale bars E,F = 15 µm.

**Figure 9:**
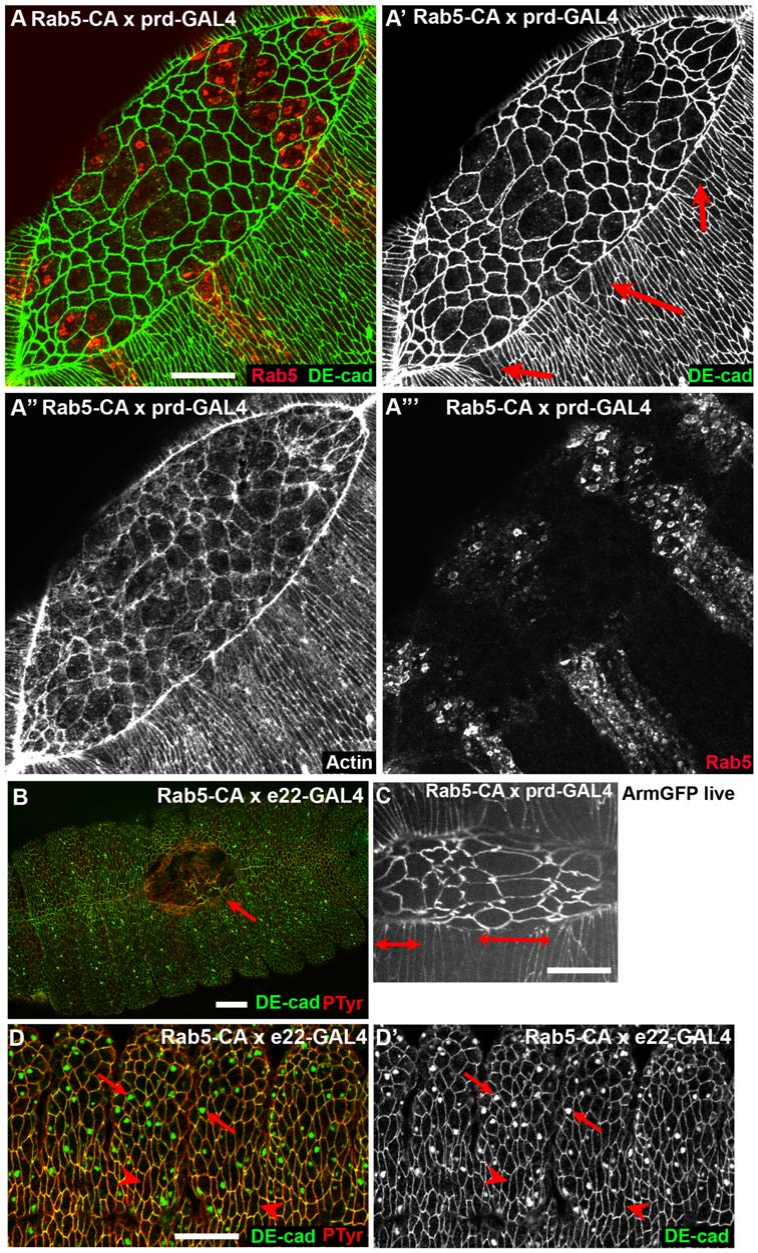
Activated Rab5 disrupts dorsal closure without dramatically altering cell surface levels of DEcad. Embryos expressing Rab5CA, antigens and genotypes indicated. A–C. Dorsal views, stage 13–14 embryos during dorsal closure. Rab5CA expression delays apical constriction of amnioserosal cells. A. Arrows, Rab5CA expressing leading edge cells are abnormally wide. B. Dorsal view, stage 14–15 embryo with defect in dorsal closure (arrow), C. Arrows, Rab5CA expressing amnioserosal cells delay apical constriction. D. Ventrolateral view, stage 14 embryo. DE-cad (but not phosphotyrosine (PTyr)) accumulates in putative vesicles (arrows) but is not reduced at AJs (arrowheads). Scale bars = 30 µm.

We compared this to the effects of constitutively activating Rab5 GTPase, by the expression of a constitutively active mutant (Rab5 Q88L; gift of M. Gonzalez-Gaitan; [54], [61]; called here Rab5CA). Activation of Rab5 by expression of Rab5CA had striking effects on DEcad localization. Endogenous Rab5 localized to puncta distributed through the cytoplasm that often co-localized with DEcad in the basal region of the ventral epidermis (Fig. 1D; Fig. 8A–D, cells not in regions bracketed by arrows, Fig. 8E–E″′). In contrast, in cells expressing Rab5-CA, Rab5 accumulated in enlarged presumptive early endosomes basal to the AJs (Fig. 8A–D, cells in regions encompassed by arrows). In the ectoderm DEcad accumulated at high levels in these putative endosomes (Fig. 8F–F″′), and Crb co-localized with DEcad in these structures (Fig. 8G–G″). These enlarged “early endosomes” also had striking “cages” of actin surrounding them (Fig. 8F–F″′). However, the enhanced accumulation of DEcad in putative endosomes did not apparently reduce cell surface levels of DEcad, either in the extended germband stage (Fig. 8A) or even much later during dorsal closure (Fig. 9A, D–D′). Despite this dramatic cell biological phenotype, most normal morphogenetic movements proceeded on schedule. However, cells expressing Rab5CA failed to complete morphogenetic changes during dorsal closure. Ubiquitous expression using e22-GAL4 disrupted the completion of dorsal closure (Fig. 9B); this was also apparent in cuticle preparations in which large dorsal holes and head holes were observed (Fig. 3N,O). The defects were even more apparent when Rab5CA was expressed using prd-GAL4 in stripes; apical constriction of amnioserosa cells expressing Rab5CA was slowed significantly relative to their non-expressing neighbors (Fig. 9A–A″′, C), and cells at the leading edge had broadened leading edges (Fig. 9A′, arrows), possibly because of the amnioserosal defect. Taken together, these data suggest that Rab5-mediated endocytosis is important for the alteration of cell shapes during dorsal closure.

## Discussion

The past twenty years have seen major advances in our understanding of the components of the adherens junction and other apical and basolateral junctional complexes, and their inter-relationships. However, this is largely a static picture that doesn't accommodate the rapid changes in cell adhesion, cell shape, and cell arrangement during embryonic and postembryonic development. One postulated contributor to junctional plasticity is traffic of junctional proteins, including both targeting after initial synthesis and endocytosis for recycling or destruction. Here we explored one aspect of this larger question, the roles of Rab11 and Rab5 in the *Drosophila* embryonic ectoderm, a model epithelium.

### Junctional proteins may be trafficked differently in different epithelia

Our data suggest that Rab11 plays a critical role in maintaining epithelial integrity in the embryonic ectoderm. Reducing its function using either a well-characterized dominant-negative or loss-of-function alleles leads to loss of epithelial integrity, due to disruption of AJs. Interestingly, the ventral ectoderm is most sensitive to perturbations in Rab11 function. This correlates well with previous work that suggests that the ventral epidermis is more sensitive to reduction in DEcad function, and that this can be alleviated by reducing morphogenesis in this tissue by preventing neuroblast invagination [47], [62].

Our data further suggest that loss of Crb precedes loss of DEcad. This is consistent with the known role of Crb in maintaining stability of AJs in the embryo [32]. It may suggest that in this tissue, Crb is the direct target of Rab11, with effects on DEcad and AJs secondary. Of course, Rab11 could also be important for trafficking of both junctional proteins, with Crb simply more sensitive to reduction.

Our data fit nicely with earlier work by Blankenship et al. [34]. They reduced the function of the exocyst protein Exo84, and found similar results. Ectodermal epithelial integrity was lost, and Crb was affected before AJ proteins or Baz. Strikingly, in Exo84 mutants, Crb and AJ proteins both accumulated in an expanded Rab11-positive recycling endosome. These data are consistent with a model in which the exocyst is important for trafficking of junctional proteins from the recycling endosome to the cell surface, either after initial synthesis or after endocytosis. They also suggest that Crb is more sensitive to this block than DEcad. Our data further support this hypothesis, as blocking Rab11 function has similar effects in junctional protein localization and on epithelial integrity. Recent work from Harris and Tepass [48] provides additional information about junctional protein trafficking in the embryonic ectoderm. Their data suggest that Cdc42, Baz, aPKC, and Par6 may negatively regulate Rab5 directed endocytic uptake of both Crb and DEcad, thus maintaining epithelial integrity. Their data are also consistent with Crb being the primary target, and demonstrate that the ventral epidermis is especially sensitive to reductions in Cdc42 function. However, while loss of Exo84, Rab11, and Cdc42 all appear to have their primary effects on Crb, the effects of these manipulations may be quite distinct, since while Rab5 blockade clearly affects endocytosis, Rab11 may be acting either in the initial biosynthetic pathway or in recycling. In the future, it will be interesting to further explore the localization of DE-cad and Crb in *Rab11* mutant cells, characterizing their accumulation at different places in the trafficking pathways, and exploring whether they are targeted for degradation. Analysis of double mutants between *Rab11* and other trafficking (e.g., *Rab5*) or protein degradation regulators would also help reveal how DE-cad and Crb are trafficked.

It is interesting to compare and contrast these results in the embryonic ectoderm with results in other epithelial tissues. In the embryonic trachea, data suggest that DEcad may be a direct target of Rab11-mediated trafficking, with regulated Rab11 function regulating cell intercalation [45]. In most cells in postembryonic imaginal discs, precursors of the adult epidermis, Crb is likely not a critical target of trafficking, as it is not essential for AJ integrity in larval imaginal discs [63]. However, junctional proteins are regulated by trafficking in imaginal discs. In the pupal wing disc, dominant negative Rab11 has effects on AJ integrity, reducing cell surface DEcad [33], reminiscent of what we observed in the embryonic ectoderm. Similar effects on DEcad and AJs were seen in the pupal eye disc [43]. The exocyst also plays an important role in maintaining cell surface DEcad in wing discs [13]. In cells with reduced exocyst function, DEcad accumulates in enlarged Rab11-positive recycling endosomes [13], as was observed in the embryonic ectoderm [34]. In wing discs, data suggests that exocyst is important for initial delivery of DEcad to the plasma membrane [13]. In wing discs and in embryos, Cdc42, Par6 and aPKC are important for restraining AJ endocytosis [48], [51], [64], but in imaginal discs the block appears to occur after accumulation in vesicles but before vesicle scission [51], [64]. One future challenge will be to explore how trafficking of distinct proteins becomes rate limiting in different tissues. This may reflect tissue specific mechanisms for regulating biosynthetic delivery or endocytic uptake of proteins like DE-cadherin—data comparing the effects of Cdc42 loss in the pupal eye [65], [66], the wing imaginal disc [51], [64] and the embryo [48]provide striking examples of these differences, though the mechanisms underlying them remain unclear.

Crb does play an important role in AJ integrity during photoreceptor differentiation [63]. In these highly polarized cells, Rab11 and the exocyst plays a key role in trafficking Rhodopsin and other apical proteins to the rhabdomere [39]–[41]. In photoreceptors, reductions in Rab11 or Sec6 function do not eliminate polarized cell architecture, but this may be due to perdurance of wild-type protein, as only small clones of mutant cells were observed.

Our data do not support as critical a role for Rab5 in the embryonic ectoderm. Expression of dominant-negative Rab5 did not disrupt AJs or epithelial integrity, although it did impede dorsal closure. It is possible, however that residual Rab5 activity may suffice in the ectoderm. Alternately, the effects of Rab11 may be on delivery of newly synthesized cadherin rather than that recycled from endocytic vesicles, accounting for the difference. Constitutively active Rab5 led to striking accumulation of DEcad and Crb in enlarged putative early endosomes, but surprisingly did not reduce levels of cell surface DEcad, suggesting that endocytic uptake is not rate limiting. This could be further tested by examining whether defects might emerge if we genetically reduced levels of DE-cad or Crb in embryos expressing constitutively active Rab5. In contrast, in both the follicular epithelium during oogenesis and the eye imaginal disc in larval development, Rab5 regulates uptake of Crb, maintaining the size of the apical compartment, although loss of Rab5 does not have strong effects on membrane levels of DEcad [31]. These data emphasize the importance of exploring junctional trafficking in different tissues, as distinct mechanisms appear to be used at different times and places to produce the diverse cell behaviors seen during development.

## Materials and Methods

### Genetics and fly stocks

Mutations and balancer chromosomes are described at FlyBase (flybase.bio.indiana.edu). Wild type was *y w*. Fly stocks used in this study are described in Table 1. All experiments were done at 25°C. In Rab11 crosses, females carrying UAS-transgenes were crossed to males with GAL4-drivers. In Rab5 crosses, males carrying UAS-transgenes were crossed to females with GAL4-drivers. Cuticle preparations were as in [67].

**Table 1:** Reagents used.

Fly Stock	Source
*y w* (wild-type)	Bloomington Drosophila Stock Center
*rab11^j2D1^*/TM3	Bloomington
*rab11^93Bi^*/TM3	Bloomington
*rab11* ^Δ*FRTi*^/TM3	R. Cohen, University of Kansas
w;UAS-Rab11-N124I/CyO-KrGFP	D. Ready, Purdue University
*shg^2^*/CyO, KrGFP	Peifer lab
*crb^2^*/TM3,TwiGFP	Bloomington
Ubi-DE-Cad-GFP (II)	H.Oda, JT Biohistory Research Hall, Osaka JAPAN
prd-Gal4,armGFP/TM6	Peifer Lab
UAS-Rab5 43N/TM3	M. Gonzalez-Gaitan, Université de Genève, SWITZERLAND
UAS-Rab5 43N/CyO	M. Gonzalez-Gaitan
w;UAS-Rab5 Q88L/TM6	M. Gonzalez-Gaitan
prd-Gal4/TM3	Bloomington
w; en-Gal4 (II)	Bloomington
e22-Gal4/CyO,KrGFP	Bloomington
Nanos-Gal4 NGT40 (III)	Bloomington
**Antibody and concentration used**	**Source**
Anti-DE-Cad (1∶100)	Developmental Studies Hybridoma Bank (DSHB)
Anti-Crb (1∶100)	DSHB
Anti-Dlg (1∶200)	DSHB
Anti-Bazooka (1∶500)	T. Harris, University of Toronto
Anti-Rab11 (1∶1000)	D. Ready
Anti-Rab5 (1∶1000)	M. Gonzalez-Gaitan
Alexa-phalloidin (1∶500)	Molecular Probes (Carlsbad, CA, USA)

### Imaging and Immunoblotting

For immunofluorescence microscopy, embryos were prepared and imaged as previously described [68]. Briefly, embryos were treated for 20 minutes in 1∶1 heptane:3.7% formaldehyde, hand-devitellinized, and blocked/stained in PBS/1% goat serum/0.1%TritonX-100. For antibodies/probes see Table 1. Secondary antibodies were Alexa-conjugates from Molecular Probes/Invitrogen and were used at 1∶1000. Sample mounting was in Aqua-Polymount (Polysciences). For fixed sample imaging, Zeiss LSM510 or Pascal confocal microscopes and LSM software were used. Images were acquired from live embryos imaged at 25°C using a spinning-disc confocal (Perkin-Elmer CSU10), a 12-bit camera, and Metamorph. Time-lapse imaging was as described previously [69]. All images were processed using Adobe Photoshop. For analysis of protein levels by immunoblotting, the homozygous *rab11* mutant embryos (20–24 hrs) were selected by hand using fluorescence microscopy, using a GFP balancer chromosome to determine genotype. Protein samples were prepared by grinding embryos on ice in Laemmli buffer with a plastic pestle and boiling 5 minutes. The blot was incubated with anti-Rab11 antibody at 1∶5000.

## Supporting Information

Video S1 DEcad::GFP is both at AJs and in vesicles in ectoderm. Stage 9–10 embryo expressing Ubi-DEcad::GFP. Note DEcad::GFP at AJs and in putative vesicles. Still is in Fig. 1B. Images were captured every 10 sec.
(7.87 MB MOV)

Video S2 DEcad::GFP is both at AJs and in vesicles in the amnioserosa. Stage 12–13 embryo expressing Ubi-DEcad::GFP,, which is both at AJs and in putative vesicles. Stills are in Fig. S2D. Images were captured every 10 sec.
(7.42 MB MOV)
